# Developmental regulation of GABAergic gene expression in forebrain cholinergic neurons

**DOI:** 10.3389/fncir.2023.1125071

**Published:** 2023-03-24

**Authors:** Adam J. Granger, Karen Mao, Jessica L. Saulnier, Morgan E. Hines, Bernardo L. Sabatini

**Affiliations:** Department of Neurobiology, Harvard Medical School, Boston, MA, United States

**Keywords:** GABAergic transmission, cholinergic transmission, neurotransmitter co-transmission, acetylcholine, GABA

## Abstract

Acetylcholine and GABA are often co-released, including from VIP-expressing neurons of the cortex, cortically-projecting neurons of the globus pallidus externus and basal forebrain, and hippocampal-projecting neurons of the medial septum. The co-release of the functionally antagonistic neurotransmitters GABA and acetylcholine (ACh) greatly expands the possible functional effects of cholinergic neurons and provides an additional exogenous source of inhibition to the cortex. Transgene expression suggests that nearly all forebrain cholinergic neurons in mice at some point in development express *Slc32a1*, which encodes the vesicular GABA transporter (VGAT). To determine the degree of co-expression of GABA and Ach handling proteins, we measured expression in adult mice of *Slc32a1*, *Gad1* and *Gad2* (which encode GAD67 and GAD65, respectively, the GABA synthetic enzymes) in cholinergic neurons using fluorescent *in situ* hybridization. We found that only a subset of cholinergic neurons express the necessary machinery for GABA release at a single time in adult mice. This suggests that GABA co-release from cholinergic neurons is dynamic and potentially developmentally regulated. By measuring expression of *Slc32a1*, *Gad1, Gad2*, and *Chat* in the basal forebrain and medial septum in mice from post-natal day 0 to 28, we noted abundant yet variable expressions of GABAergic markers across early development, which are subsequently downregulated in adulthood. This is in contrast with the forebrain-projecting pedunculopontine nucleus, which showed no evidence of co-expression of GABAergic genes. These results suggest that expression of GABA signaling machinery in the cortically-projecting cholinergic system peaks during early development before settling at a non-zero level that is maintained through adulthood.

## Introduction

Acetylcholine (ACh) is a major neuromodulator in the mammalian forebrain that regulates higher brain functions including attention and memory. These cognitive functions of ACh are primarily mediated through cholinergic signaling in the cortex and hippocampus (Ballinger et al., 2016), where the primary sources of ACh are long-range projections from cholinergic neurons in the nucleus basalis and medial septum, respectively (Mesulam, 1995). Acetylcholine influences cortical and hippocampal circuits through excitatory, ionotropic nicotinic receptors (nAChRs) (Gotti et al., 2006), and through muscarinic metabotropic receptors (mAChRs) whose effects are dependent on the specific receptor subtype. Specifically, M1-type mAChRs are coupled to G_*q*_ and increase excitability in downstream neurons, whereas M2-type mAChRs are G_*i*_-coupled, and can act as autoreceptors to inhibit pre-synaptic release (Thiele, 2013). Though the effects of ACh signaling are complex, it is generally considered an excitatory neuromodulator that gates the flow of activity in the cortex and promotes synaptic plasticity.

We have previously shown that nearly all cholinergic neurons of the mouse forebrain express the cellular machinery necessary to release the inhibitory neurotransmitter GABA, including those that project to the cortex from the nucleus basalis, and hippocampal-projecting neurons of the medial septum (Saunders et al., 2015a; Granger et al., 2016). Likewise, optogenetic activation of cholinergic neurons results in inhibitory post-synaptic currents in cortical neurons (Saunders et al., 2015a,b). The synaptic co-transmission of GABA introduces additional complexity to the potential circuit effects of signaling from cholinergic neurons and an additional source of inhibition to the cortex and hippocampus.

Co-transmission of GABA from cholinergic neurons has since been described in a variety of forebrain cholinergic cell populations. These include from a subpopoulation of cortical interneurons that express VIP and ChAT which differentially release ACh and GABA onto distinct post-synaptic targets (Granger et al., 2020). Activation of cholinergic neurons in the medial prefrontal cortex of mice and rats also provides a strong cholinergic output to neighboring neurons (Obermayer et al., 2019), though these neurons are likely not VIP-expressing and therefore it is unclear if they also release GABA (Granger et al., 2020). Co-transmission of GABA and Ach from distinct vesicles can also be elicited from cholinergic projections from the medial septum to the hippocampus, where GABA effectively suppresses local network activity (Takács et al., 2018). Outside of the cortex and hippocampus, GABA co-transmission has been demonstrated from a subset of striatal cholinergic interneurons (Lozovaya et al., 2018), and from laterodorsal tegmental nucleus onto dopaminergic neurons of the substantia nigra (Estakhr et al., 2017; Le Gratiet et al., 2022). Prior to any demonstration of ACh/GABA co-transmission in the forebrain, the differential release of these two transmitters from starburst amacrine cells of the retina has been shown to establish light direction selectivity in retinal ganglion cells (Lee et al., 2010; Sethuramanujam et al., 2016).

In many cases, co-transmission of multiple neurotransmitters is a dynamic and regulated process. Neurons may switch which neurotransmitter they release throughout development, in response to stress or toxicity, or as a result of experience (Spitzer, 2015; Li et al., 2020; Steinkellner et al., 2022). Synapses may also shift the relative contribution of multiple neurotransmitters to post-synaptic responses, as is the case in GABA/glutamate co-transmitting synapses in the lateral habenula (Shabel et al., 2014; Meye et al., 2016). Similarly, cholinergic transmission from GABAergic neurons of the lateral septum changes over time, with an increasing proportion of GABAergic neurons demonstrating labeling with cholinergic markers when allowed to integrate over longer time periods (Hunt et al., 2022). Although this evidence shows that regulation of neurotransmitter identity is a common feature of co-transmitting neurons, whether GABA release from forebrain cholinergic neurons is dynamic is unknown.

In this study, we investigated the regulation of GABAergic gene expression in forebrain cholinergic neurons in mice throughout development, focusing particularly on neurons in the nucleus basalis, which provides a major cholinergic input to the cortex, and the medial septum, which provides cholinergic input to the hippocampus. We found that while a cumulative genetic labeling of GABAergic markers marks all cholinergic neurons as GABA-releasing, only a subset of cholinergic neurons express GABAergic markers at a particular time in adult mice. This co-expression was dynamic, peaking during the 1st and 2nd postnatal weeks before decreasing to lower levels in adulthood. Finally, this co-expression was not found in the forebrain-projecting cholinergic neurons of the pedunculopontine nucleus (PPN), suggesting a specific function for basal forebrain cholinergic neurons.

## Materials and methods

### Animals

For genetic labeling of GABAergic markers, we used mice expressing Cre recombinase from the endogenous locus for *Slc32a1* (Tong et al., 2008), provided by Brad Lowell (Beth Isreal Deaconess Medical Center), currently available from Jackson labs (Bar Harbor, ME; *Slc32a1^tm 2(cre)Lowl^/*J, stock #: 016962, referred to here as *Slc32a1^ires–Cre^*). These mice were crossed to the Ai6 reporter line which expresses zsGreen in the presence of Cre recombinase (Madisen et al., 2010), also available from Jackson labs (B6.Cg-*Gt(ROSA)26Sor^tm 6(CAG–ZaGreen1)Hze^*/J, stock #: 007906, referred to here as *Rosa26^lsl–zsGreen^*). Wild-type C57BL/6 mice of both sexes were used for fluorescent *in situ* hybridization of medial septum and basal forebrain, and pedunculopontine nucleus. All animal care and experimental manipulations were performed in accordance with protocols approved by the Harvard Standing Committee on Animal Care, following guidelines described in the US NIH *Guide for the Care and Use of Laboratory Animals*.

### Fluorescent in situ hybridization sample preparation and imaging

Mice were anaesthetized through isoflurane inhalation, except for mice at P7 and younger were anesthetized on ice. Cerebral hemispheres were removed, and immediately frozen in cold 2-methylbutane solution. The frozen brains were subsequently embedded in OCT-containing cryomolds and kept in –80°C ultra-freezer until sample sectioning.

Before sectioning, the frozen OCT-embedded brains were equilibrated at –20°C overnight. The cryomold was peeled out of the OCT compound block. 20 μm thick sections were prepared on cryostat Leica CM3050 S and mounted on VWR micro slides Superfrost Plus slides. These sections were stored at −80°C for up to two weeks before processing. Samples were fixed with 4% paraformaldehyde and stained according to the ACD RNAscope Fluorescent Multiplex Assay manual. Sections were incubated at room temperature for 30 s with DAPI. Excess liquid was removed and immediately coverslipped with ProLong antifade reagent. Antisense probes for *ChAT, Slc32a1*, *Gad1*, and *Gad2* were purchased from Advanced Cell Diagonstics (ACD).^1^ Images of nucleus basalis, medial septum and pedunculopontine nucleues were obtained with a Keyence BZ-X710 microscope using a 60X oil immersion objective.

### Immunohistochemistry sample preparation and imaging

Mice were anaesthetized by isoflurane inhalation and trans-cardially perfused with PBS followed by 4% PFA in PBS. Brains were stored in 4% PFA in PBS for at least 8 h at 4°C. Brains were sliced into 50 μm thick sections with a Leica VT1000s vibratome. Selected slices were transferred to a clean twelve-well plate and blocked at room temperature for an hour with shaking in blocking buffer (10% normal goat serum, Abcam), 0.25% TritonX-100 in PBS. Blocking buffer was removed and replaced with 1 ml of 1:100 α-ChAT primary antibody (Sigma, AB144P) in blocking buffer. Slices were incubated with the primary antibody overnight at 4°C with gentle shaking. The next day, slices were transferred to a clean well and washed six times, 5 min each in PBS. Following the final wash, slices were incubated for 2 h in 1:500 donkey α-goat secondary antibody conjugated to Alexa Fluor 594 dye (ThermoFisher, A-11058) diluted in blocking buffer. Slices were washed six times in PBS (5 min for each wash) before mounting with DAPI (Thermo Fisher Scientific) and imaged on a VS120 slide scanning microscope (Olympus) with a 10x objective and a Keyence BZ-X710 microscope using a 60X oil immersion objective.

### Image analysis and data analysis

A custom MatLab (MathWorks) script was written to quantify colocalization of cholinergic and GABAergic markers. First, ROIs were semi-automatically drawn around areas of *in situ* labeling for *Chat* to define *Chat*^+^ cells. The boundary was set to have a 0.25 convexity to ensure proper approximation of *Chat*^+^ cells. The images were analyzed for proportion coverage, or the ratio of fluorescent pixels in each optical channel to the total pixels in a cellular ROI. After background subtraction and fluorescence thresholding in all channels using Otsu’s method, the proportion coverage in *ChAT*, *Slc32a1* (encoding VGAT/VIAAT) and *Gad1,2* (encoding GAD67 and GAD65, respectively) channels were measured within the *Chat*^+^ cellular ROIs. All images underwent thresholding using Otsu’s method with no other manipulations. These data were used to generate X-Y plots displaying the percent coverage for each channel per ROI as well as the cumulative distribution across all cells for coverage by each marker. For direct comparison across timepoints, ROIs with the proportion coverage of a GABAergic marker higher than 0.025 were defined as positive. The percentage of *Chat*^+^ cells positive for *Slc32a1*, *Gad1,2* and both were calculated to generate bar graphs on Python. Statistical parameters including the value of n, measurements of arithmetic mean and standard error of the mean (mean ± SEM), were reported in the figures and the figure legends. Sample data and custom scripts are publicly available on GitHub^2^.

### Statistical analysis

Bar graphs with error bars represent the mean ± standard error of the mean. Significance is indicated by asterisks, with * meaning *p* ≤ 0.05, ^**^ meaning *p* ≤ 0.01, and ^***^ meaning *p* ≤ 0.001. All statistical analysis was carried out in MatLab (MathWorks). Significant difference in the distributions of each cell’s coverage by *Chat, Slc32a1*, and *Gad1,2* across developmental stages (Figure 2B and Supplementary Figure 1) was calculated with a one-way ANOVA with Bonferroni correction for multiple comparisons. Differences in the proportion of *ChAT*^+^ cells that are positive for *Slc32a1*, *Gad1,2*, or co-positive for both (Figures 2C–H). were calculated using Student’s t-test, with comparisons between each developmental stage and adulthood, adjusted using Bonferonni correction for multiple comparisons.

## Results

**FIGURE 1:**
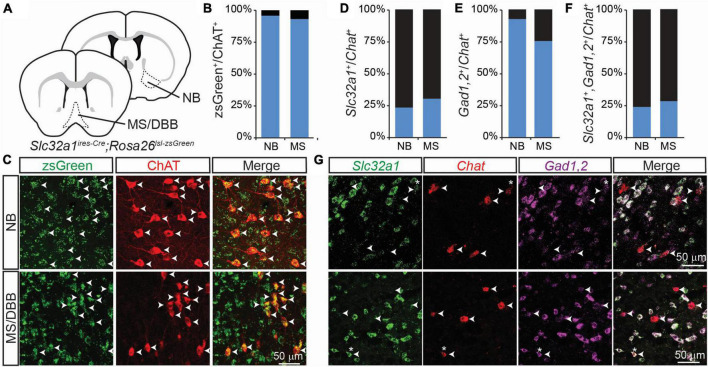
Integrated genetic labeling shows higher co-expression of GABAergic markers in cholinergic cells than fluorescent *in situ* hybridization in adult mice. **(A)** Schematic showing the brain regions imaged for this study, including the nucleus basalis (NB), which provides the main cholinergic input to the cortex, and medial septum/diagonal band of broca (MS/DBB), which provides cholinergic input to the hippocampus. Genetic labeling of *Slc32a1* expression is achieved by crossing *Slc32a1^ires–Cre^* mice, which expresses Cre recombinase from the endogenous *Slc32a1* locus, with *Rosa26^lsl–zsGreen^*, which expresses the soma-localizing fluorophore zsGreen in a Cre-dependent fashion. **(B,C)** Quantification and example images of zsGreen-expressing neurons co-labeled with an immunostain for ChAT (598 zsGreen^+^/624 ChAT^+^ NB neurons from n = 3 mice; 560 zsGreen^+^/601 ChAT^+^ MS/DBB neurons from *n* = 3 mice. Data adapted from Saunders et al. (2015a). **(D–F)** Quantification of colocalization of FISH for *Slc32a1* **(D)**, *Gad1,2* **(E)** or both **(F)** with *Chat*, (49 *Slc32a1*^+^/199 *Chat*^+^ BF neurons from *n* = 5 mice and 54 *Slc32a1*^+^/181 *Chat*^+^ MS neurons from n = 4 mice; 159 *Gad1,2*^+^/199 *Chat*^+^ BF neurons from n = 5 mice and 86 *Gad1,2*^+^/181 *Chat*^+^ MS neurons from *n* = 4 mice; 48 co-positive/*Chat*^+^ 199 BF neurons from *n* = 5 mice and 52 co-positive/181 *Chat*^+^ MS neurons from n = 4 mice). **(G)** Example images showing FISH for *Slc32a1* (green), *Chat (red*), *Gad1*,2 (Magenta), and the merged imaged. Arrow heads show the positions of *Chat*^+^ neurons, and asterisks indicate *Chat*^+^ ROIs with strong co-expression of *Slc32a1* and *Gad1,2*.

In a previous study (Saunders et al., 2015a), we examined the co-expression of GABAergic markers in cholinergic forebrain neurons using a genetic strategy in which neurons that express *Slc32a1*, the gene encoding the vesicular GABA transporter/vesicular inhibitory amino acid transporter (VGAT/VIAAT), are labeled by the fluorophore zsGreen – specifically we examine the distribution of zsGreen in *Slc32a1^ires–Cre^*;*Rosa26^lsl–zsGreen^* mice. We found that nearly 100% of neurons in the forebrain that stained for choline acetyltransferase (ChAT), which synthesizes ACh, also expressed zsGreen (Figures 1A–C). We saw similar results when we labeled for expression of *Gad2*, which encodes the GABA synthetic enzyme GAD65 (Saunders et al., 2015a, *Gad2^ires–Cre^*;*Rosa26^lsl–zsGreen^*). This suggests that all forebrain cholinergic neurons, including those in the nucleus basalis (NB) and medial septum/diagonal band of broca (MS/DBB), are capable, at some point in development, of synthesizing and releasing GABA.

However, this genetic strategy provides an integrated, cumulative labeling of all neurons that express these GABAergic markers at any point during development and does not indicate which cells express these markers at any given individual time. To address this issue, we used fluorescent *in situ* hybridization (FISH) to measure the co-expression of *Chat*, *Slc32a1*, and a combined label of both *Gad1* and *Gad2* in 2-3 month old mice. In contrast to the genetic labeling strategy, we found that only a small proportion of *Chat*^+^ cells also expressed *Slc32a1* (Figures 1D, G), which we have previously shown to be necessary for GABA release from cholinergic neurons (Saunders et al., 2015a). A larger portion of *Chat*^+^ neurons co-expressed *Gad1* or *Gad2* (Figures 1E, G), and nearly all *Chat*^+^ neurons that expressed *Slc32a1* also expressed *Gad1,2* (Figures 1F, G), indicating that expression of the vesicular GABA transporter is the limiting factor for GABA release from cholinergic neurons.

**FIGURE 2:**
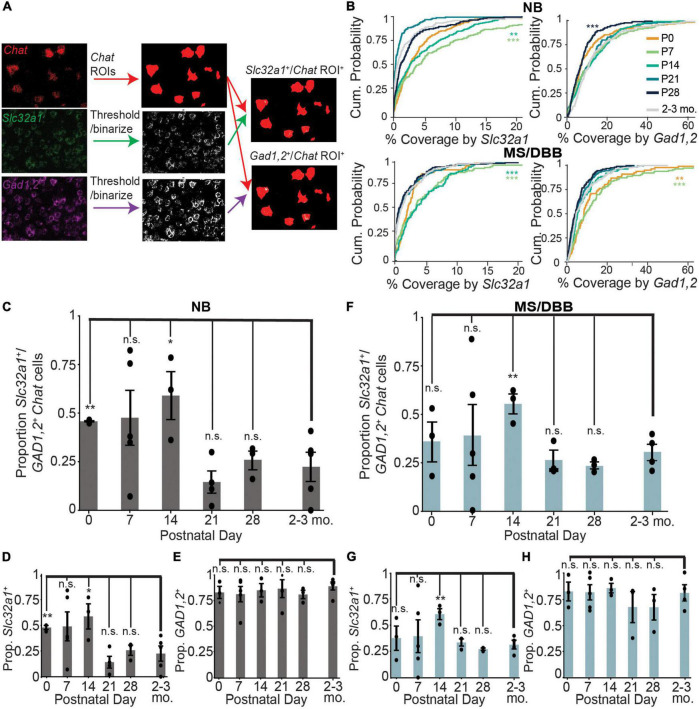
Expression of *Slc32a1* and *Gad1,2* peak at postnatal days 7 and 14 in *ChAT*^+^ neurons of the NB and MS/DBB. **(A)** Schematic of the imaging analysis pipeline. Raw images of *ChAT* fluorescence are binarized and converted to ROIs corresponding to *Chat*-expressing neurons. *Slc32a1* and *Gad1,2* signal are thresholded and binarized, and the puncta overlap with *Chat* ROIs (yellow) and quantified for percent coverage. **(B)** Cumulative distribution of *Chat* ROI coverage by *Slc32a1* (left panels) and *Gad1,2* (right panels) expression across all neurons in the NB (top row) and MS/DBB (bottom row) between P0 and P28. Asterisks indicate statistically significant differences in the distribution of ROI coverage at each developmental time point compared to cells from 2-3 month old mice (**p ≤ 0.01, ***p ≤ 0.001). **(C–E)** Quantification of *Chat*^+^ cells in NB that are co-positive for both *Slc32a1* and *Gad1,2* **(C)**, *Slc32a1* alone **(D)**, or Gad1,2 alone **(E)**. **(F–H)** Quantification of *Chat*^+^ cells in MS/DBB that are co-positive for both *Slc32a1* and *Gad1,2* **(F)**, *Slc32a1* alone **(G)**, or Gad1,2 alone **(H)**. Data from **(B–H)** includes between 73-319 *Chat*^+^ cells from either BF or MS/DBB, from *n* = 3-5 mice per time point. Data are represented as mean ± s.e.m., Asterisks indicate statistically significant differences in the proportion of positive cells in each mouse compared to 2-3 month old mice (*p ≤ 0.05, **p ≤ 0.01).

The discrepancy between the number of co-labeled GABAergic/cholinergic neurons using an integrated genetic label versus labeling at a single timepoint using FISH suggests that GABAergic gene expression is dynamic in cholinergic neurons. To test whether expression of GABAergic markers in cholinergic neurons changes during development, we measured the expression of *Chat, Slc32a1*, and *Gad1,2* in the nucleus basalis and medial septum through development, from postnatal day 0 (P0) to 28 (P28). We measured the amount GABAergic marker expression in each *Chat*^+^ cell across development, using the *Chat* mRNA signal to define ROIs corresponding to individual cholinergic neurons (Figure 2A). We observed that expression of *Slc32a1* and *Gad1,2* is higher at early developmental time points, peaking by P14 before decreasing to levels similar to those observed in adult (2-3 month old) mice by P28. This trend holds true for both NB and MS/DBB, although expression of *Gad1,2* is higher overall than that of *Slc32a1* (Figure 2B). In contrast, expression of *Chat* increases from P0 to P14, and remains high at P28 (Supplementary Figure 1). We used this expression data to define individual *Chat*^+^ neurons as *Slc32a1*^+^ and/or *Gad1,2^+^*, as defined by a minimum of 2.5% of the cell ROI covered by RNA signal. This threshold was chosen because it defines 98% of *Chat*-defined ROIs as *Chat*^+^. By this definition, ∼50% of *Chat*^+^ neurons in the NB are positive for both *Slc32a1* and *Gad1/2* at P0, increasing to ∼60% at P14 before dropping to ∼25% by P28 (Figure 2C), which is consistent with the values we observed in 2-3 month old mice. This pattern is determined by expression of *Slc32a1* (Figure 2D), as the proportion of *Chat*^+^ cells that were positive for *Gad1,2* was stable at above 80% (Figure 2E), and nearly all *Slc32a1*-expressing cells also expressed *Gad1,2*. A similar pattern of expression was observed in the medial septum with *Gad1,2*^+^ cells stable above 60% (Figures 2F–H). Notably, compared to later timepoints, the co-expression of GABAergic markers was highly variable at P7 and P14, with some mice showing much higher co-expression than others. This suggests that not only is the expression of GABAergic markers in cholinergic neurons developmentally regulated, but that the timing is transient and may vary from mouse to mouse.

**FIGURE 3:**
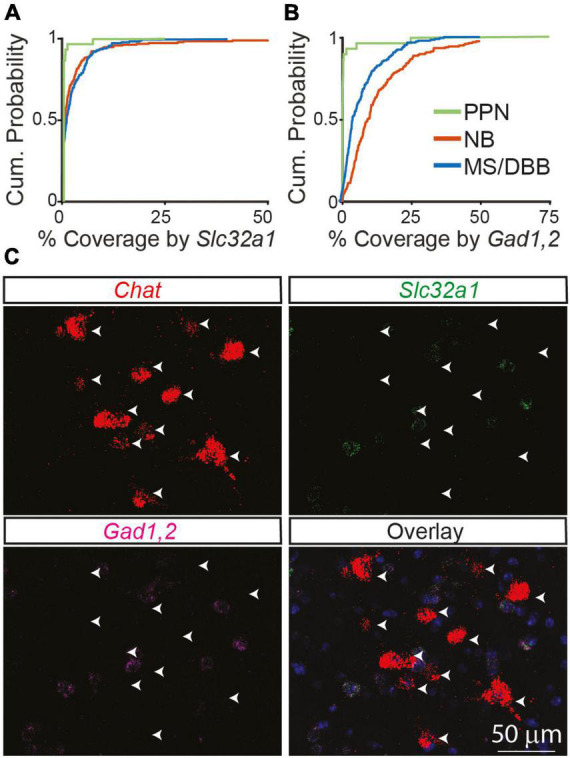
GABAergic markers are not expressed in cholinergic neurons in the PPN of adult mice compared to the NB and MS/DBB. **(A)** Cumulative distribution of *Chat* ROI coverage in the PPN by *Slc32a1* **(A)** and *Gad1*,2 **(B)** compared to neurons in the NB and MS/DBB. Data from PPN includes 188 *Chat*^+^ cells from *n* = 3 mice, from NB includes 199 *Chat*^+^ neurons from *n* = 5, and from MS/DBB includes 181 *Chat*^+^ cells from *n* = 4 mice. **(C)** Example images of *Chat, Slc32a1, and Gad1,2* in the pedunculopontine nucleus. Arrowheads indicate the position of *Chat*^+^ neurons across the images.

Even at developmental times with relatively low co-expression, very few neurons had no expression of *Slc32a1*. Instead, in most neurons at least some *Slc32a1* fluorescence could be observed, although often not to a degree to confidently label that neuron as *Slc32a1* expressing. To determine if this low level of signal is an artifact of our analysis methods or results from a true low-level of expression inherent to cholinergic neurons, we imaged cholinergic neurons in the pedunculopontine nucleus, in which no ChAT-labeled neurons showed genetic labeling for GABAergic markers (Saunders et al., 2015a). We find that expression of *Slc32a1* is lower in the *Chat*-expressing neurons of the PPN than even many of the *Slc32a1* “negative” cells we identify in NB and MS/DBB from adult 2-3-month old mice (Figure 3A), indicating that this signal can neither be attributed purely to background fluorescence or to an inherent “leak” of *Slc32a1* expression in all cholinergic neurons. The difference is starker when considering *Gad1,2*, as most *Chat*^+^ NB and MS/DBB neurons in adult mice have detectable *GAD1,2* expression whereas almost no *Chat*^+^ PPN neurons do (Figures 3B, C). The comparison with PPN highlights that the expression of GABAergic markers in NB and MS/DBB cholinergic neurons, even when present at low levels, represents a *bona fide* biological property of these cells.

## Discussion

In this study, we find that the co-expression of the GABAergic markers *Slc32a1* and *Gad1,2* is higher in cholinergic neurons of the NB and MS/DBB during early post-natal development, suggesting that GABA co-transmission from these neurons is also elevated during this period. Similar co-expression of these markers could not be found in neurons of the PPN, which is a midbrain cholinergic nucleus that sends projections throughout the forebrain. Many neurons that we classify as lacking *Slc32a1* expression in adult mice (defined as below 2.5% coverage of the cell) in the NB and MS/DBB had higher *Slc32a1* signal than neurons in the PPN, and *Gad1,2* expression in NB and MS/DBB remained high throughout development and adulthood. These data show that expression of GABAergic genes is a consistent phenotype of forebrain cholinergic neurons that changes throughout development, although what levels of expression are required for co-transmission of GABA remains unclear.

*Gad1*,2 expression is uniformly higher than Slc32a1 at all observed timepoints. One potential reason why this might be the case is that GAD proteins can often provide purely metabolic functions in the cell which may not be related to the co-transmission of GABA (Soghomonian and Martin, 1998). However, because we did not observe GAD expression in the PPN, it is likely not essential for cholinergic neuron function. Instead, we propose that NB and MS/DBB cholinergic neurons are “primed” to become GABAergic with high baseline levels of GAD protein to synthesize GABA. The neurons can then modulate expression *Slc32a1* to produce VGAT/VIAAT and increase the degree of GABA co-transmission. Alternatively, it is possible that *Slc32a1* is a very stable protein and that occasional expression of this gene is sufficient to maintain vesicular packaging of GABA and functional GABAergic synapses. Further studies are required to test these hypotheses.

The elevated co-expression observed during development only partially explains the discrepancy we observed between the nearly 100% co-labeling of GABAergic markers using genetic strategies compared to ∼25% observed using FISH in adult mice. The greatest degree of co-expression observed by FISH was at most 60%, with highly variable expression at P7 and P14. One possibility is that peak co-expression occurs between P7 and P14, and we missed a transient period of synchronous co-expression. Another possibility is that cholinergic neurons asynchronously increase GABAergic gene expression, such that eventually nearly all express GABAergic genes at some point. Given the variability we observed at P7 and P14, we cannot rule out the possibility that some unidentified factor or stimulus in addition to development can regulate expression of GABAergic genes.

The function of inhibition by GABA co-transmission from cholinergic neurons in the cortex or hippocampus remains unknown. Cre-driven ablation of GABA co-transmission at ACh synapses elicits significant alterations in social, spatial and fear memory; our findings suggest that such effects may result from a cumulative disruption involving all basal forebrain cholinergic neurons, masking the functionality of GABA co-transmission at a specific timepoint (Goral et al., 2022). The timing during which we observed peak co-expression of GABAergic and cholinergic markers coincides with periods of peak synaptogenesis in the cortex, raising the possibility that GABA release may play some role in establishing appropriate cholinergic synaptic connectivity. In the cortex, GABAergic synapses typically form before excitatory glutamatergic synapses (Owens et al., 1999). In addition, the reversal potential of chloride through GABA_*A*_ receptors is often depolarizing in immature neurons (Ben-Ari et al., 2012), which can activate voltage-gated calcium channels and promote synapse maturation (Pfeffer et al., 2009). Altering the reversal potential of GABA to be hyperpolarizing in immature neurons disrupts the formation of cortical circuits, and the same may be true of cholinergic projections.

Although decreasing into adulthood, a small population of NB and MS/DBB neurons retain robust expression of both *Slc32a1* and *Gad1* or *Gad2* in adult mice. The function of this signaling is also unknown and may be distinct from the more widespread signaling that occurs during development. Because ACh and GABA have antagonistic post-synaptic effects via ionotropic receptors, the net effect of cholinergic signaling may be complex. We previously showed that a subpopulation of cortical VIP interneurons that express ChAT can differentially release ACh and GABA onto separate post-synaptic targets (Granger et al., 2020), allowing for synergistic effects of the two transmitters in the cortex. Further studies are required to determine if ACh and GABA release are similarly segregated in projections from NB and MS/DBB. Finally, it is also unknown if the strong Slc32a1 expression in the ∼25% of cholinergic neurons in adults is stable, or whether these neurons shift between purely cholinergic and a dual ACh/GABA co-transmitting state. Regardless, GABA co-transmission from cholinergic neurons provides an additional layer of complexity to cholinergic signaling in the forebrain that is only beginning to be understood.

## Data availability statement

The raw data supporting the conclusions of this article will be made available by the authors, without undue reservation.

## Ethics statement

This animal study was reviewed and approved by the Harvard Medical Area Standing Committee on Animals.

## Author contributions

AG and BS conceived of the study. KM, MH, and JS gathered all the data. AG and KM performed analysis of the data. AG, KM, and BS wrote the manuscript with comments and feedback from the other authors. All authors contributed to the article and approved the submitted version.

## Footnotes

1
http://acdbio.com/

2
https://github.com/bernardosabatinilab
