# Supplementary material for: Developmental regulation of GABAergic gene expression in forebrain cholinergic neurons
Source: Front Neural Circuits. 2023 Mar 24;17:1125071. doi: 10.3389/fncir.2023.1125071 (PMC10080005; doi:10.3389/fncir.2023.1125071)
Supplement: Supplementary file 1 [file Data_Sheet_1.docx]

Supplementary Material

Developmental regulation of GABAergic gene expression in forebrain cholinergic neurons

Adam J Granger^1^†^*^, Karen Mao^1^†, Jessica L Saulnier^1^, Morgan E Hines^1^, Bernardo L Sabatini^1^


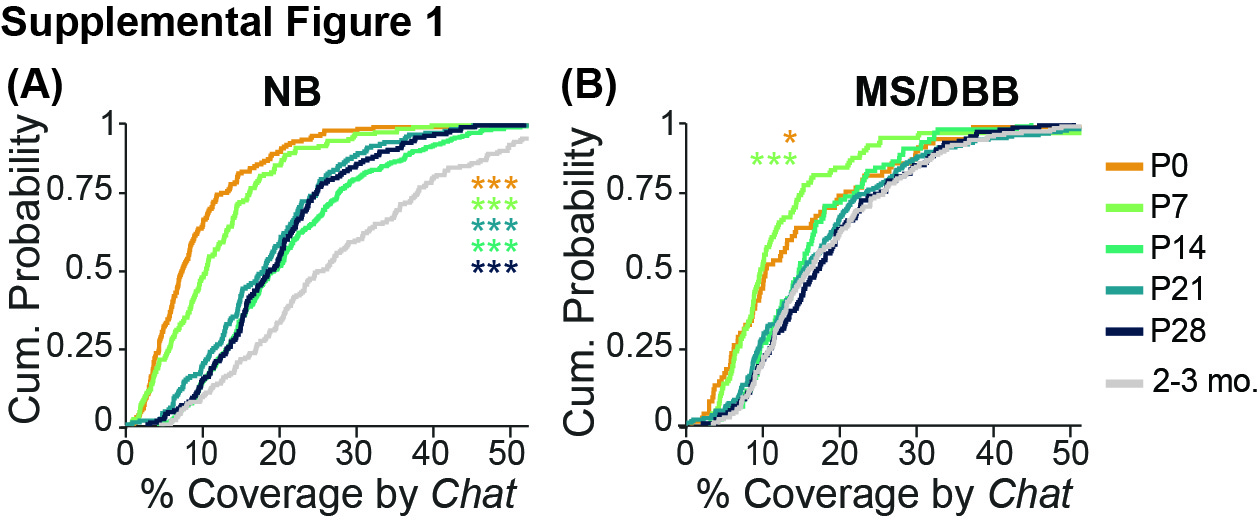
*** Correspondence:** Adam J Granger: adam_granger@hms.harvard.edu

**Supplemental Figure 1: Expression of *ChAT* increases between P0 and P28.** Cumulative distribution of Chat ROI coverage by *Chat* signal in the NB (A) and MS (B). Because ROIs are determined by the boundaries of *Chat* signal, these plots both provide a minimum signal coverage that can be used to determine if a cell positively expresses a given mRNA. 98% of *Chat* ROIs have greater than 2.5% coverage by *Chat* fluorescence. *Chat* coverage increases from P0 to P14 without decreasing, as is the case for *Slc32a1* and *Gad1,2*. Data from A & B includes between 73-163 *Chat*^+^ from n = 3-5 mice per time point. Asterisks indicate statistically significant differences in the distribution of ROI coverage at each developmental time point compared to cells from 2-3 month old mice (* = p ≤ 0.05. *** = p ≤ 0.001).
